# Stereochemistry of ring-opening/cross metathesis reactions of *exo*- and *endo*-7-oxabicyclo[2.2.1]hept-5-ene-2-carbonitriles with allyl alcohol and allyl acetate

**DOI:** 10.3762/bjoc.11.204

**Published:** 2015-10-13

**Authors:** Piotr Wałejko, Michał Dąbrowski, Lech Szczepaniak, Jacek W Morzycki, Stanisław Witkowski

**Affiliations:** 1 Institute of Chemistry, University of Białystok, ul. Ciołkowskiego 1K, 15-245 Białystok, Poland

**Keywords:** Grubbs’ catalysts, metathesis, ROCM, ROMP, Z-selectivity

## Abstract

The ROCM reactions of *exo*- and *endo*-2-cyano-7-oxanorbornenes with allyl alcohol or allyl acetate promoted by different ruthenium alkylidene catalysts were studied. The stereochemical outcome of the reactions was established. The issues concerning chemo- (ROCM vs ROMP), regio- (1-2- vs 1-3-product formation), and stereo- (*E*/*Z* isomerism) selectivity of reactions under various conditions are discussed. Surprisingly good yields of the ROCM products were obtained under neat conditions.

## Introduction

Substituted tetrahydrofurans are a common motif found in many biologically active natural products [1–2], e.g., annonaceous acetogenins [3–4], lignans [5–6], iso- and neurofurans [7–8], as well as macrodiolides [9]. These substances exhibit a diverse biological activities including antitumor, antimicrobial, etc. [10–12].

**Scheme 1:**
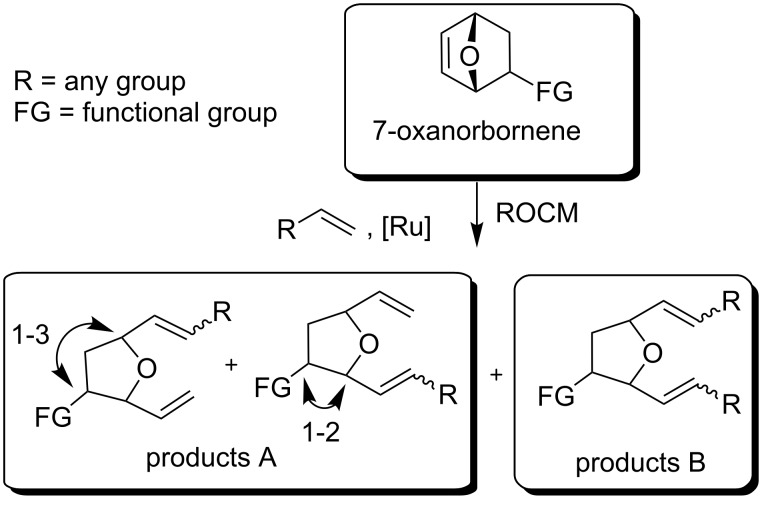
ROCM reactions of 7-oxanorbornene.

**Figure 1:**
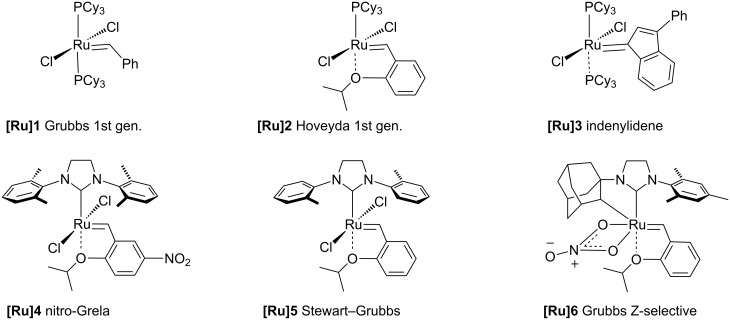
Chemical structures of catalysts **[Ru]1–6** used in this work.

Stereoselective construction of substituted tetrahydrofurans is still a challenging task in natural product synthesis [13–17]. One of the most promising approaches to solve this problem seemed to be the metathetic opening of substituted 7-oxanorbornenes, which was first developed by Blechert and co-workers [18–19]. They started with the ring-opening of strained alkenes (mostly 7-oxanorbornene) followed by cross metathesis with a cross partner (e.g., propene) to give the respective ring-opening cross metathesis (ROCM) products. Preliminary analysis suggested that these transformations lead mainly to incorporation of two molecules of a coupling partner, if present in excess, into tetrahydrofurans to give a product of type B. Blechert has reported that the incorporation of only one unit of a terminal alkene (Scheme 1; products A) was also possible using only a slight excess of a terminal alkene [19]. Arjona et al. have noticed that when 7-oxanorbornenes bearing a bulky C2-substituent are used in ROCM, products of type A are formed in higher yields and with good regioselectivities [20]. Treatment of 2-acetoxy-7-oxanorbornene (Scheme 1; FG = OAc) with allyl acetate in the presence of **[Ru]1** (Figure 1) catalyst afforded mainly the product A of a 1–3 type (75% yield, 1-3:1-2 = 4:1), while the 2-hydroxy derivative (FG = OH) provided an equimolar ratio of both type A products. The authors have suggested that the observed regioselectivity comes from steric effects that favour formation of 1-3 over 1-2 metallacycles in the former case (see [20]).

The completely regioselective ROCM of 2-tosyl (FG = Ts) substituted 7-oxanorbornene was reported by Rainier [21]. The *endo* substrate gave only the regioisomer of a 1-2 type (*E*/*Z*, 1:1), whereas the *exo* substrate yielded a mixture of products of types 1-2 and 1-3 (9:1; *E*/*Z* 0.9:1).

Arjona, Blechert and others have suggested that a competing ring-opening metathesis polymerization (ROMP) can be minimized by carrying out the reaction in high dilution. Furthermore good yields of ROCM products can be obtained only when an 1.5-fold excess of a cross olefin is used [14,19–20].

## Results and Discussion

Now, we wish to report our preliminary results of ROCM reactions of *exo*- and *endo*-7-oxabicyclo[2.2.1]hept-5-en-2-carbonitrile (**1** and **2,** respectively) with allyl acetate (**3**) or allyl alcohol (**4**) catalyzed by several commercially available ruthenium catalysts (**[Ru]1–6**, Figure 1). To the best of our knowledge there is no example of a ROCM reaction of 7-oxanorbornene bearing the –CN substituent with olefins. However, Arjona and co-workers described closely related transformation of 7-oxanorbornenes (bearing carbonyl, OH and ether substituents [20]) but any detailed information about the influence of the reaction conditions on the product ratio 1-2 vs 1-3 and geometry *Z*/*E* was reported. It should be emphasized that in the presence of a nitrile group an efficient metathetic transformation is difficult to carry out due to a competitive complexation of Ru by the nitrile group [22]. The influence of the reaction conditions on the distribution of the type A products and their *E*/*Z* stereochemistry was studied. Our results seem to be in some contradiction to the commonly accepted opinion that ROCM reactions should be carried out in high dilution to avoid polymerization. It was found that ROCM reactions proceed quite efficiently even under neat conditions. Furthermore, less complex mixtures of products were formed and they were easier to separate from the ROMP products.

**Scheme 2:**
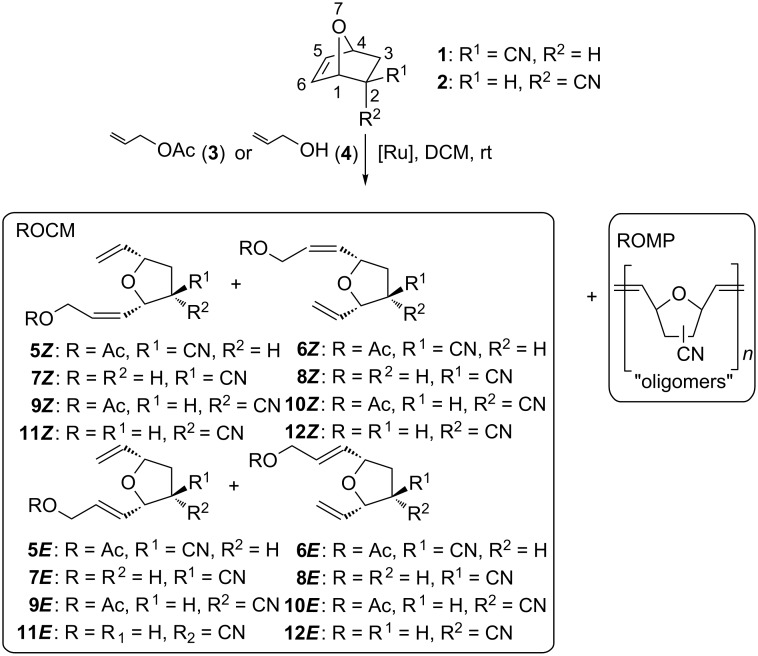
Metathesis products of *exo*- and *endo*-7-oxabicyclo[2.2.1]hept-5-en-2-carbonitriles (**1** and **2**) with allyl acetate (**3**) or allyl alcohol (**4**).

7-Oxanorbornenes **1** and **2** were treated with olefin **3** or **4** in the presence of ruthenium catalysts **[Ru]1–6** (Figure 1) to afford mixtures of tetrahydrofurans **5**–**12** (Scheme 2). The mixtures were carefully separated using PTLC techniques. The pure samples of compound **6*E***, **7*Z***, **8*Z***, **8*E***, **10*E*** and **12*Z*** were isolated and characterized spectroscopically (^1^H and ^13^C NMR, GC–MS).

Two types of the regioisomeric products of type A should be taken into consideration (Scheme 1). The distinguishing of 1-2 from 1-3 isomers was done by analysis of coupling constants ^3^*J*_H,H_ or ^3^*J*_C,H_ in 2D NMR (DQF and HMBC) experiments, respectively. The *E*/*Z* geometry of the double bonds were determined on the basis of ^3^*J*_CH=CH_ constants from an ^1^H experiment recorded with ^1^H-homodecoupling or with *J*-resolved techniques [23]. The compounds **6*E*** and **10*E*** were deacetylated (MeOH/KCN) [24] to give **8*E*** and **12*E***. The samples **7*Z***, **8*Z***, **8*E***, and **12*Z*** were subjected to acetylation (Py/Ac_2_O) to give **5*Z***, **6*Z***, **6*E*** and **10*Z***, respectively. The acetates were directly characterized by GC–MS. Based on retention indices and literature data (MS spectra identity of *E*/*Z* isomers) [25], the identification of all ROCM components of the aforementioned mixtures was performed.

**Figure 2:**
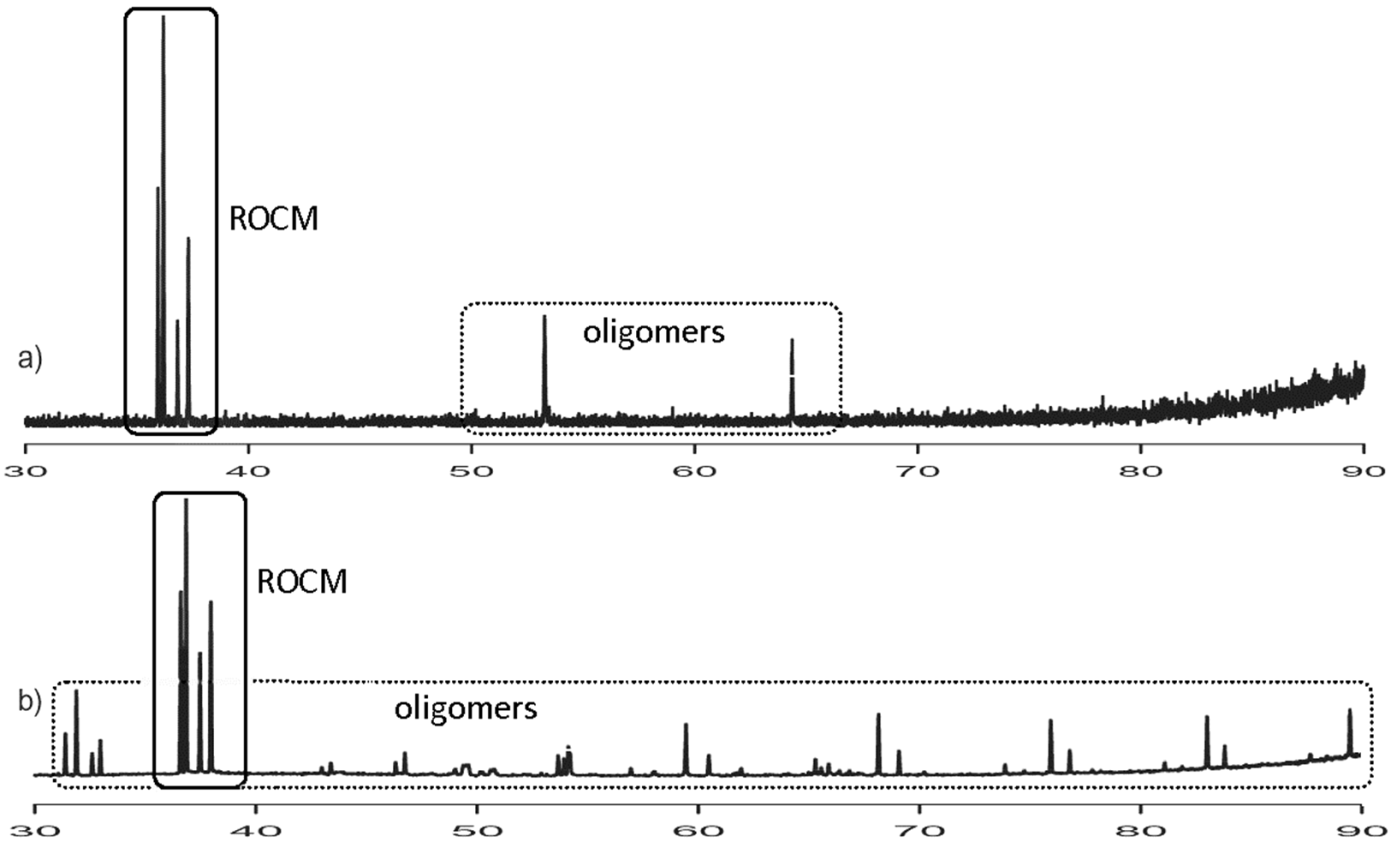
Representative GC chromatograms recorded from crude reaction mixtures described in Table 1: a) entry 1 and b) entry 5.

The results of these experiments are given in Table 1 and Table 2. The collected data show that the reactions in diluted solutions (Table 1, entries 1, 7, 10, and 13) led mainly to ROMP products, whereas ROCM products were formed in lower yields. However, according to literature data, the formation of a ROMP product can be minimized by carrying out the reactions in high dilution [18,20–21]. In our case, the experiments carried out in more concentrated solutions (Table 1, entries 3, 4, 8 and 12) gave substantially higher yields of ROCM products. Our results seem to be in contradiction to those reported by Blechert [15] and Arjona [20]. Satisfactory results were obtained even when the reactions were carried out under neat conditions (Table 1, entries 5, 6, 9, and 12). Furthermore, the resulted mixtures were easier to work-up and to separate from ROMP oligomers by simple filtration (see Figure 2).

**Table 1:** Results of ROCM reactions of nitriles **1** and **2** with olefins **3** and **4**.

reagents/conditions				products/ratio^a^							

entry	alkenes	molar ratio^b^	conc.^c^ (mol/L)	total yield (%)	**5*Z*** (1630)^d^	**5*E*** (1654)^d^	*E*/*Z*	**6*Z*** (1637)^d^	**6*E*** (1667)^d^	*E*/*Z*	*1-2*/*1-3* (**5**:**6**)

1	**1**+**3**	1:1	0.023	30 (67)^e^	23	11	32:68	45	21	32:68	34:66
2	**1**+**3**	1:1	0.115	33 (45)^e^	22	10	31:69	46	22	32:68	32:68
3	**1**+**3**	1:1	0.575	57 (33)^e^	21	12	36:64	45	22	33:67	33:67
4	**1**+**3**	1:10	0.115	67 (21)^e^	23	12	34:66	44	20	31:69	35:65
5	**1**+**3**	1:10	neat	65	23	10	30:70	46	21	31:69	33:67
6	**1**+**3**	1:20	neat	58	24	10	29:71	46	20	30:70	34:66

					**7*Z*** (1503)^d^	**7*E*** (1548)^d^	*E*/*Z*	**8*Z*** (1528)^d^	**8*E*** (1551)^d^	*E*/*Z*	1-2/1-3 (**7:8**)

7	**1**+**4**	1:1	0.023	59	22	21	49:51	26	31	54:46	43:57
8	**1**+**4**	1:1	0.115	65	21	20	49:51	31	28	47:53	41:59
9	**1**+**4**	1:10	neat	56	19	19	50:50	33	29	47:53	38:62

					**9*Z*** (1676)^d^	**9*E*** (1713)^d^	*E*/*Z*	**10*Z*** (1712)**^d^**	**10*E*** (1738)^d^	*E*/*Z*	1-2/1-3 (**9**:**10**)

10	**2**+**3**	1:1	0.023	35 (40)^e^	17	5	23:77	52	26	33:67	22:78
11	**2**+**3**	1:1	0.115	38 (37)^e^	14	5	26:74	52	28	35:65	19:81
12	**2**+**3**	1:10	0.115	70 (25)^e^	10	5	33:67	57	28	33:67	15:85

					**11*Z*** (1575)^d^	**11*E*** (1583)^d^	*E*/*Z*	**12*Z*** (1602)^d^	**12*E*** (1614)^d^	*E*/*Z*	1-2/1-3 (**11**:**12**)

13	**2**+**4**	1:1	0.023	36	21	10	32:68	38	31	45:55	31:69
14	**2**+**4**	1:1	0.115	38	13	8	38:62	48	31	39:61	21:79
15	**2**+**4**	1:10	neat	46	20	17	46:54	32	30	48:52	37:63

^a^Conditions: **[Ru]1**, 5 mol %, DCM, rt, 18–24 h; percentage contents of products in mixtures based on the intensity of GC–MS signals; ^b^molar ratio of 7-oxanorbornene **1** or **2** to the olefin; ^c^concentration of **1** or **2** in DCM (mol/L); ^d^the retention indices; ^e^isolated yield of ROMP products (*n* = 2–9).

**Table 2:** Influence of the catalyst on ROCM product distribution (reaction of **1** with **3**)^a^.

entry	catalyst	products/ratio^b^						

		**5*Z***	**5*E***	***E***/***Z***	**6*Z***	**6*E***	***E***/***Z***	1-2/1-3 (**5**:**6**)

1	**[Ru]1**	23	12	34:66	44	20	31:69	35:65
2	**[Ru]2**	20	14	41:59	44	23	34:66	34:66
3	**[Ru]3**	20	13	39:61	45	22	33:67	33:67
4	**[Ru]4**	20	17	46:54	37	26	41:59	37:63
5	**[Ru]5**	13	25	66:34	24	37	61:39	38:62
6	**[Ru]6**	24	11	31:69	55	10	15:85	35:65

^a^Conditions: 1 equiv of **1**, 10 equiv of allyl acetate (**3**), 5 mol % of catalyst **[Ru]1–6**, rt, 18–24 h (0.115 M of **1** in DCM); ^b^percentage contents of products in the mixtures based on the intensity of GC–MS signals.

A different isomeric products distribution was observed in the mixtures of type A products (1-2 and 1-3). In reactions of *exo*- and *endo*-norbornene **1** and **2**, with the acetate **3**, approximately a two-fold excess of the 1-3 *Z* isomers was formed, and the least abundant product among the four diastereoisomers was the 1-2 *E* isomer (5–12% relative yield; see Table 1). In reactions of **1** with **4** the amount of each product in the mixture (Table 1, entry 8) was in the range of 20% to 31%. However, the total ratio of 1-2 vs 1-3 products in most experiments was the same (ca. 1:2), except entries 10, 11, 12, and 14, where the portion of the 1-3 regioisomers was higher (in entry 11 even 1:4). According to Arjona et al. [20] the observed regioselectivity comes from the steric hindrance of the –CN group, however, some electronic effects in 7-oxanorbornene should be also taken into consideration. Unexpectedly, the *Z*-selectivity predominated in most reactions catalyzed by **[Ru]1** (see Table 1, entries 1–6, 10–15, and Table 2, entries 1–4). A two-fold excess of the *Z* isomer was observed in both groups of products (1-2 and 1-3). While the reaction of *endo*-nitrile **2** with alcohol **4** proceeded with lower *Z*/*E* selectivity (1.5:1) (Table 1, entries 13–15), the *exo*-isomer **1** reacted without any stereoselectivity (Table 1, entries 7–9).

It is worth to note that the reactions of nitriles **1** and **2** promoted by the Grubbs *Z*-selective catalyst **[Ru]6** (Table 2, entry 6) in anhydrous THF provided a fraction of ROCM products only in 14% yield (by GC – complete substrate conversion) with regioselectivity similar to that observed for the Grubbs I catalyst (**[Ru]1**) (Table 2, entry 1). In general the more reactive catalysts, namely Grubbs II and Hoveyda–Grubbs II, favored the formation of ROMP products. A different distribution of *E/Z*-isomers was observed in the reaction of substrate **1** with alcohol **3** in the presence of the Steward–Grubbs (**[Ru]5**) catalyst (Table 2, entry 5). The *E*-isomers of both products **5** and **6** prevailed, while the 1-2 vs 1-3 ratio was almost the same as those in other experiments. It should be noted that less sterically demanding *o-*tolyl-N-substituents in NHC-ligands provide more space around the ruthenium center.

**Scheme 3:**
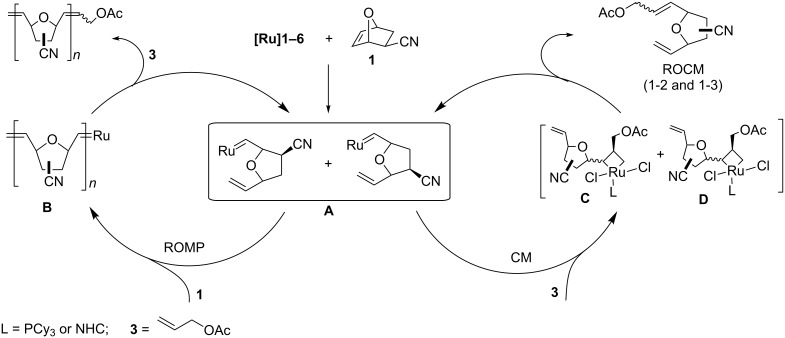
The plausible mechanism of the formation of ROCM and ROMP products from *exo-* or *endo-*7-oxabicyclo[2.2.1]hept-5-ene-2-carbonitriles **1** or **2**. For simplicity of the scheme, the reaction of only *exo-*stereoisomer **1** as a substrate is presented.

In general, ROCM reactions are most successful when highly strained substrates are used. Furthermore, this transformation should be considered as a two-step reaction where the ring-opening metathesis (ROM) is the initial step followed by a CM. It is well known that oxanorbornenes (e.g., **1** and **2**) are generally excellent substrates for ROCM reactions [14]. The cycloaddition of the ruthenium carbene **[Ru]** to a cyclic alkene **1** or **2** affords a metallacyclobutane of the 1-2 or 1-3 type (Scheme 3). Accordingly to Arjona et al. [20] a preference of the 1-3 structures over 1-2 arises from the steric interaction between the metal–ligand moiety and the substituent at position C-2 of 7-oxanorbornene. However, an influence of the electron density of the C=C bond in the starting material, as well as its complexation effects, cannot be ruled out. In reactions of **1** (*exo*) the observed 1-2/1-3 ratio varied from 1:1.4 in to 1:2, while **2** (*endo*) gave a much higher content of the 1-3 isomer, from 1:3.5 to 1:5.6. The *endo*-face metallacyclobutane was proposed as a main intermediate [26]. Decomposition of the 1-2 and 1-3 intermediates leads to ring-opened alkylidenes **A**, which can react further in two different ways, depending on the reaction speed ratio of **A** with the strained substrate **1** or **2** (ROMP) and with the terminal olefin **3** or **4** (ROCM). This step seems to be crucial for the selectivity of the ROCM, which competes with the ROMP metathesis. It is worth to note that the reaction of **A** in diluted solutions (0.023 mol/L) with the starting olefin was faster than that with the terminal olefin. As a consequence the ROMP products prevailed. On the other hand the formation of polymeric products **B** may be suppressed by using the olefinic cross partner in excess and increasing concentration of reagents (the best results were obtained in the neat experiments). It should be noted that a different *E*/*Z* selectivity was observed for experiments with **[Ru]1–4** compared to that of **[Ru]5**. In our opinion, the likely explanation of this fact is a different interaction between ligand moiety and the two substituents connected to metallacyclobutane intermediates (Scheme 3, **C** or **D**). According to Fortman and Nolan [27] the di-*N*,*N*’-*o*-tolyl substituted NHC ligand in **[Ru]5** exerts a smaller steric effect than the -PCy_3_ residue in **[Ru]1–4**. Furthermore, the bulky phosphine ligand (PCy_3_) expands away from the transition metal center (coordination sphere), while the *N*,*N*’-*o*-tolyl substituent attached to the central imidazole ring penetrates the coordination sphere. Connon and Blechert [15] suggested that a difference in energy between metallacyclobutane intermediates influences stereochemistry of metathetic products. In our case, more bulky catalysts (**[Ru]1–4**) prefer formation of *Z*-isomers in excess because the intermediate **C** is less strained than **D**. On the other hand, an interaction of less bulky *o*-tolyl ligands with the ruthenium core in **[Ru]5** causes an opposite selectivity (*E* preference). One can assume that the observed change in the *E*/*Z* selectivity resulted from chelating and electronic effects in postulated intermediates **C** and **D**. It is clear, that the *E*/Z selectivity depends on the catalyst applied, while the regioselectivity is largely substrate-dependent. The application of the ROCM products in the synthesis of natural products will be reported in due course.

## Conclusion

The ROCM reactions of 2-cyano-7-oxanorbornenes with allyl alcohol and allyl acetate may be partially stereocontrolled by a proper choice of the reaction catalyst. However, the regioselectivity largely depends on the starting material structures. Unexpectedly, the chemoselectivity of the ROCM product formation in competition with the undesired ROMP reaction may be improved by using neat reaction conditions.

## Experimental

**Figure 3:**
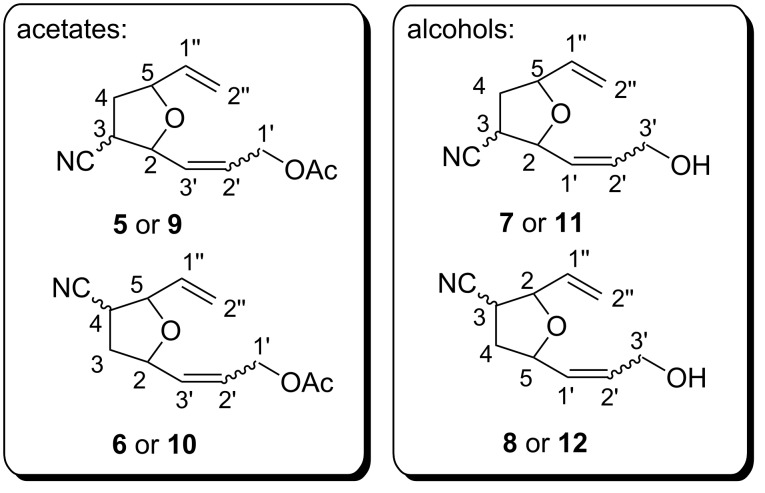
Numbering of carbon atoms in cross metathesis products.

A mixture of **1** and **2** (1.6:1) is readily available from the Diels–Alder reaction of furan and acrylonitrile according to the literature procedure [28–29]. The pure isomers were isolated by column chromatography. The ^1^H and ^13^C NMR spectra were identical with those described in the literature [30–31]. Compounds **5**–**12** were numerated based on auto name option in ChemBioDraw v. 13.0 (Figure 3).

^1^H and ^13^C NMR spectra for CDCl_3_ solutions were obtained using a Bruker Avance II spectrometer (400 and 100 MHz, respectively). Chemical shifts (δ) are reported in ppm downfield from TMS. The assignment of chemical shifts in solution was supported by 2D NMR experiments (DFQ, HSQC and HMBC). GC–MS was carried out on an Agilent Technologies HP 6890 N gas chromatograph with mass selective detector MSD 5973 (Agilent Technologies, USA). The device was fitted with a ZB-5MSi fused silica column (30 m × 0.25 mm i.d., 0.25 μm film thickness), with electronic pressure control and split/splitless injector. Helium flow rate through the column was 1 mL/min in a constant flow mode. The injector worked at 250 °C in split (1:50) mode. The initial column temperature was 50 °C rising to 340 °C at 3 °C/min and the higher temperature was maintained for 15 min. The MS detector acquisition parameters were as follows: transfer line temperature equalled to 280 °C, MS Source temperature to 230 °C and MS Quad temperature to 150 °C. The EIMS spectra were obtained at 70 eV of ionization energy. The MS detector was set to scan 40–600 a.m.u. After integration, the fraction of each component in the total ion current was calculated. Retention indices (RI) were calculated according to the formula proposed by van Den Dool and Kratz [25] with *n*-alkanes as references substances. RI values for phases type DB-5 and MS spectra for derivatives **5**–**12**, realized at an ionization energy of 70 eV are shown in Supporting Information File 1.

### General ROCM procedure

To a mixture of norbornene **1** or **2** and alkene (for details see Table 1 and Table 2) in anhydrous DCM a solution of catalyst **[Ru]1–6** (5 mol %) in DCM was added to obtain a final concentration of **1** or **2** in CH_2_Cl_2_ (0.023, 0.115 or 0.575 mol/L). For neat experiments (Table 1, entries 5, 6, 9 and 15) solid catalyst was used. The resulting mixture was stirred overnight at rt, then 0.5 mL of vinyl ethyl ether was added, and the reaction mixture was stirred for 10 min. The solvent was removed under reduced pressure and then the residue was redissolved in DCM and filtered through a pad of Celite. The crude reaction mixtures were purified by MPLC and PTLC chromatography. The structures of isolated compounds were determined by ^1^H and ^13^C NMR.

**(*E*)-3'-(4-Cyano-5-vinyltetrahydrofuran-2-yl)allyl acetate (6*E*). **^1^H NMR (CDCl_3_, δ, ppm) 5.85–5.72 (m, 3H, H-3’, 2’, 1”), 5.51, 5.47, 5.34, 5.31 (4 x ~s, 2H, H-2”), 4.60–4.56 (m, 3H, H-2 and 1’), 4.43 (dd, ^3^*J*_H,H_ = 6.72 and 7.40 Hz, 1H, H-5), 2.83–2.77 (m, 1H, H-4), 2.46–2.39 (m, 1H, H-3*eq*), 2.16–2.08 (m, 1H, H-3*ax*), 2.07 (s, 1H, CH_3_); ^13^C NMR (CDCl_3_, δ, ppm) 170.5 (C=O), 134.5 (1’), 132.2 (2’), 127.2 (1”), 119.4 (CN), 119.1 (2”) 83.1 (5) 78.5 (2), 63.7 (1’), 36.2 (3), 34.2 (4), 20.8 (CH_3_); DQF COSY (CDCl_3_) H-4 and H-3*ax*, H-4 and H-3*eq*, H-5 and H-4, H-3 and H-2; HMBC (CDCl_3_) H-2” and C-5, H-5 and C-2”, H-2 and C-3’, H-2 and C-2’; RI: 1667 (*t*_R_ = 37.34 min); TOF MS ES^+^: 244 [M + Na]^+^; HRMS *m*/*z*: [M + Na]^+^ calcd for C_12_H_15_NO_3_Na: 244.0944; found: 244.0943.

**5-((*E*)-3’-Hydroxyprop-1’-en-1’-yl)-2-vinyltetrahydrofuran-3-carbonitrile (8*E*). **^1^H NMR (CDCl_3_, δ, ppm) 5.98–5.83 (m, 2H, H-1” and 2’), 5.77–5.72 (m, 2H, H-1’), 5.51, 5.47, 5.34, 5.31 (4 x ~s, 2H, H-2”), 4.63–4.56 (m, 1H, H-5), 4.47–4.43 (m, 1H, H-2), 4.20–4.19 (m, 2H, H-3’), 2.86–2.80 (m, 1H, H-3), 2.45–2.40 (m, 1H, H-4*eq*), 2.16–2.12 (m, 1H, H-4*ax*); ^13^C NMR (CDCl3, δ, ppm) 134.6 (1’), 132.7 (1”), 129.1 (2’), 119.5 (CN), 119.1 (2”), 83.1 (2), 78.9 (5), 62.56 (3’), 36.3 (4), 34.3 (3); DQF COSY (CDCl_3_) H-3 and H-4*ax*, H-3 and H-4*eq*, H-3 and H-2, H-5 and H-4, H-5 and H-1’Hz. *J*-resolved; ^1^H NMR (CDCl_3_) ^3^*J*_H1’,H2’_ = 16 Hz; RI: 1551 (*t_R _=* 33.49 min); TOF MS ES^+^: 202 [M + Na]^+^; HRMS *m*/*z*: [M + Na]^+^ calcd for C_12_H_15_NO_3_Na 202.0844; found: 202.0848.

**5-((*Z*)-3'-Hydroxyprop-1'-en-1'-yl)-2-vinyltetrahydrofuran-3-carbonitrile (8*Z*). **^1^H NMR (CDCl_3_, δ, ppm) 5.86–5.77 (m, 3H, H-1’, 2’ and 1”), 5.51, 5.46, 5.34, 5.31 (4 x ~s, 2H, H-2”), 4.91–4.80 (m, 1H, H-2), 4.46–4.43 (m, 1H, H-5), 4.31–4.23 (m, 2H, H-3’), 2.85–2.80 (m, 1H, H-3), 2.48–2.42 (m, 1H, H-4*eq*), 2.18–2.10 (m, 1H, H-4*ax*); ^13^C NMR (CDCl_3_, δ, ppm) 134.5, 133.0, 130.2 (1’, 1”, 2’), 119.5 (CN), 119.1 (2”), 83.2, 74.8 (2 and 5), 58.8 (3’), 36.8 (4), 34.5 (3); *J*-resolved ^1^H NMR (CDCl_3_) ^3^*J*_H1’,H2’_ = 10 Hz; RI: 1529 (*t_R _=* 32.59 min); TOF MS ES^+^ *m*/*z*: [M + Na]^+^ 179, found 202; HRMS *m*/*z*: [M + Na]^+^ calcd. for C_12_H_15_NNaO_3_ 202.0844; found: 202.0849.

**2-((*Z*)-3’-Hydroxyprop-1’-en-1’-yl)-5-vinyltetrahydrofuran-3-carbonitrile (7*Z*). **^1^H NMR (CDCl_3_, δ, ppm) 5.95–5.79 (m, 2H, H-1”, 2’), 5.60–5.55 (m, 1H, H-1’), 5.36, 5.32, 5.24, 5.21 (4 x ~s, 2H, H-2”), 4.87–4.80 ( m, 1H, H-2), 4.59–4.51 (m, 1H, H-5), 3.36–3.30 (m, 1H, H-3’), 2.79–2.77 (m, 2H, H-3), 2.49–2.42 (m, 1H, H-4*eq*), 2.18–2.14 (m, 1H, H-4*ax*); ^13^C NMR (CDCl_3_, δ, ppm) 136.4 (1’), 134.7 (1”), 128 (2’), 119.5 (CN), 117.4 (2”), 79.8, 78.2 (2 and 5), 58.8 (2’), 36.3 (4), 34.6 (3); DQF COSY (CDCl_3_) H-3 and H-4*ax*, H-3 and H-4*eq*, H-5 and H-4, H-5 and H-1”, H-2 and H-1’; *J*-resolved ^1^H NMR (CDCl_3_) ^3^*J*_H1’,H2’_ = 11 Hz; RI: 1504 (*t_R _=* 31.62 min); TOF MS ES^+^: *m*/*z* 202 [M + Na]^+^; HRMS *m*/*z*: [M + Na]^+^ calcd for C_12_H_15_NO_3_Na 202.0844; found: 202.0849.

**(*E*)-3-(4-Cyano-5-vinyltetrahydrofuran-2-yl)allyl acetate (10*E*). **^1^H NMR (CDCl_3_, δ, ppm) 6.10–6.00 (m, 1H, H-1”), 6.01–5.87 (m, 2H, H-3’ 2’), 5.51, 5.47, 5.44, 5.41 (4 x ~s, 2H, H-2”), 4.65–4.59 (m, 2H, H-1’), 4.47–4.42 (m, 1H, H-2 and 5), 3.29–3.24 (m, 1H, H-4), 2.59–2.53 (m, 1H, H-3*eq*), 2.52–2.05 (m, 1H, H-3*ax*), 2.09 (s, 3H, CH_3_); ^13^C NMR (CDCl_3_, δ, ppm) 170.6 (C=O), 133.7, 132.3, 127.5 (3’, 1”, 2’), 117.7 (CN), 119.9 (2”), 80.2, 74.7 (2 and 5), 63.7 (1’), 36.7 (3), 34.4 (4), 20.8 (CH_3_); DQF COSY (CDCl_3_): H-3 and H-2, H-4 and H-5, H-1” and H-2”, H-5 and H-1”, H-2 and H-3’; *J*-resolved ^1^H NMR (CDCl_3_) ^3^*J*_H1’,H2’_ = 15–16 Hz; RI: 1738 (*t*_R_ = 39.85 min); TOF MS ES^+^ *m*/*z* [M + Na]^+^ 244; HRMS *m*/*z*: [M + Na]^+^ calcd. for C_12_H_15_NO_3_Na 244.0949; found: 244.0942.

**5-((*E*)-3’-Hydroxyprop-1’-en-1’-yl)-2-vinyltetrahydrofuran-3-carbonitrile (12*Z*). **^1^H NMR (CDCl_3_, δ, ppm): 6.10–6.00 (m, 1H, H-1”), 6.01–5.87 (m, 2H, H-1’ 2’), 5.51, 5.47, 5.44, 5.41 (4 x ~s, 2H, H-2”), 4.85–4.79 (m, 1H, H-2), 4.47–4.42 (m, 1H, H-5), 4.31–4.20 (m, 2H, H-3’), 3.29–3.24 (m, 1H, H-4), 2.59–2.53 (m, 1H, H-3*eq*), 2.52–2.05 (m, 1H, H-3*ax*), 2.09 (s, 3H, CH_3_); ^13^C NMR (CDCl_3_, δ, ppm) 170.6 (C=O), 133.6 (1”), 132.6 (2’), 130.9 (3’), 120.0 (2”), 118.9 (CN), 80.2 (5), 58.8 (1’), 37.3 (3), 34.5 (4); DQF COSY (CDCl_3_) H-3 and H-2, H-4 and H-5, H-1” and H-2”, H-2 and H-3’, H-5 and H-1” Hz; *J*-resolved; ^1^H NMR (CDCl_3_) ^3^*J*_H1’,H2’_ = 11 Hz. RI: 1583 (*t_R _=* 34.92 min); TOF MS ES^+^ *m*/*z* [M + Na]^+^ 202; HRMS *m*/*z*: [M + Na]^+^ calcd for C_12_H_15_NO_3_Na 202.0844; found: 202.0850.

## Supporting Information

File 1 MS spectra and retention indices of all compounds **5**–**12**.
