# Supplementary material for: Stereochemistry of ring-opening/cross metathesis reactions of exo- and endo-7-oxabicyclo[2.2.1]hept-5-ene-2-carbonitriles with allyl alcohol and allyl acetate
Source: Beilstein J Org Chem. 2015 Oct 13;11:1893–901. doi: 10.3762/bjoc.11.204 (PMC4661012; doi:10.3762/bjoc.11.204)

Supporting Information

for

Stereochemistry of ring-opening/cross metathesis reactions of *exo*- and *endo*-7-oxabicyclo[2.2.1]hept-5-ene-2-carbonitriles with allyl alcohol and allyl acetate

Piotr Wałejko*, Michał Dąbrowski, Lech Szczepaniak, Jacek W. Morzycki and Stanisław Witkowski

Address: Institute of Chemistry, University of Białystok, ul. Ciołkowskiego 1K, 15-245 Białystok, Poland

Email: Piotr Wałejko - pwalejko@uwb.edu.pl

*Corresponding author

MS spectra and retention indices of all compounds 5–12

5*Z*: (*Z*)-3'-(3-Cyano-5-vinyltetrahydrofuran-2-yl)allyl acetate

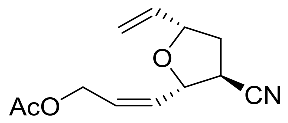

Formuła: **C12H15NO3**

MolWt: **221.1048**

Retention index: **1630**

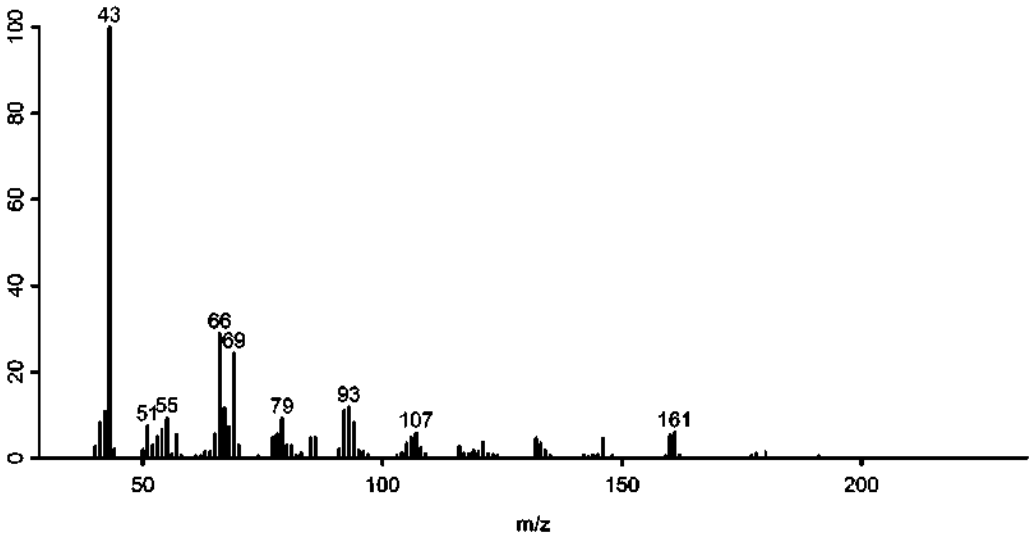

5*E*: (*E*)-3'-(3-Cyano-5-vinyltetrahydrofuran-2-yl)allyl acetate

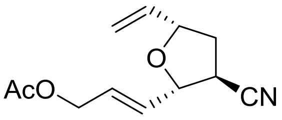

Formuła: **C12H15NO3**

MolWt: **221.1048**

Retention index: **1653**

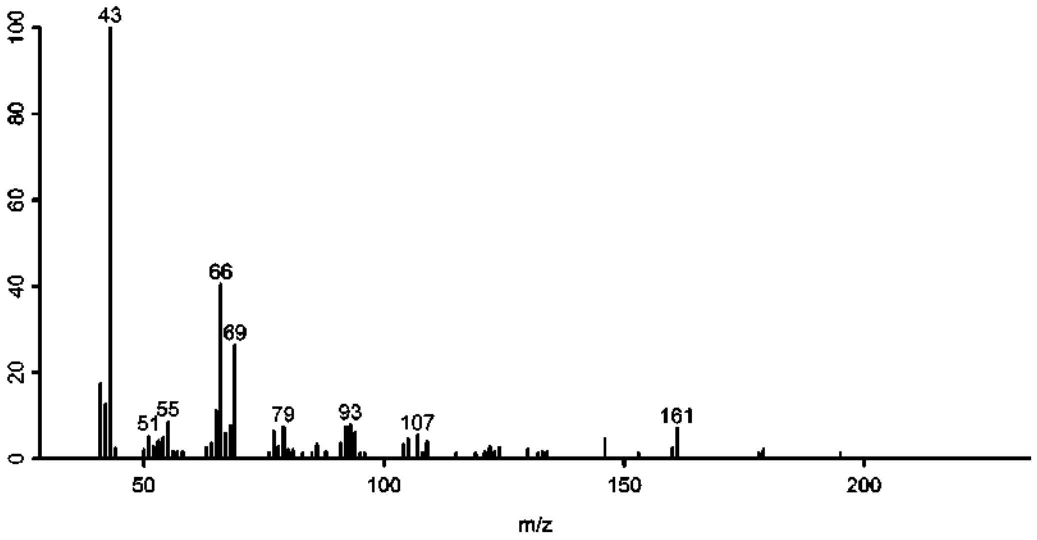

6*Z*: (*Z*)-3'-(4-Cyano-5-vinyltetrahydrofuran-2-yl)allyl acetate

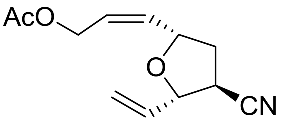

Formuła: **C12H15NO3**

MolWt: **221.1048**

Retention index: **1636**

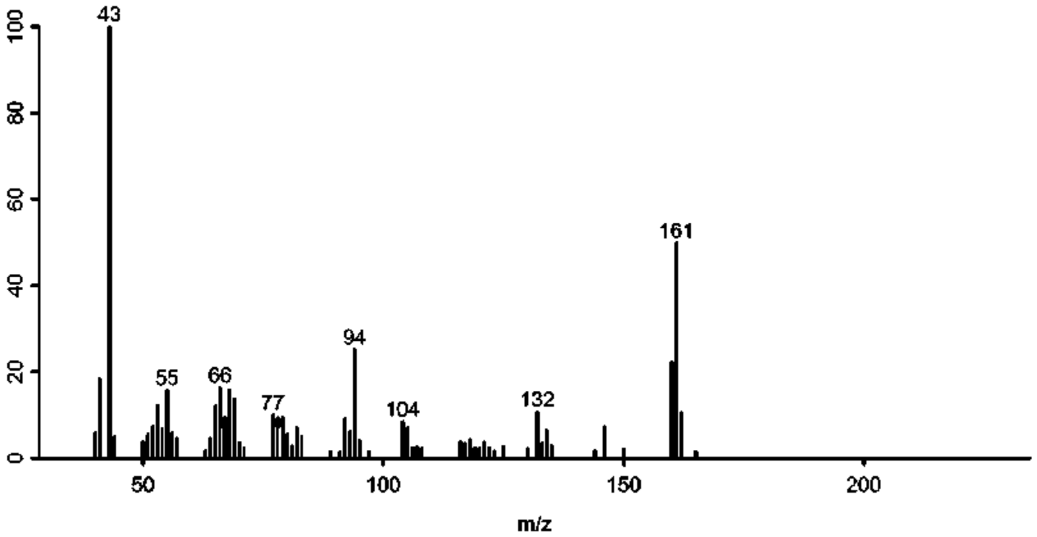

6*E:* (*E*)-3’-(4-Cyano-5-vinyltetrahydrofuran-2-yl)allyl acetate

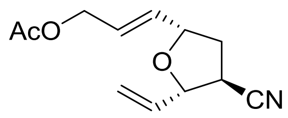

Formuła: **C12H15NO3**

MolWt: **221.1048**

Retention index: **1666**

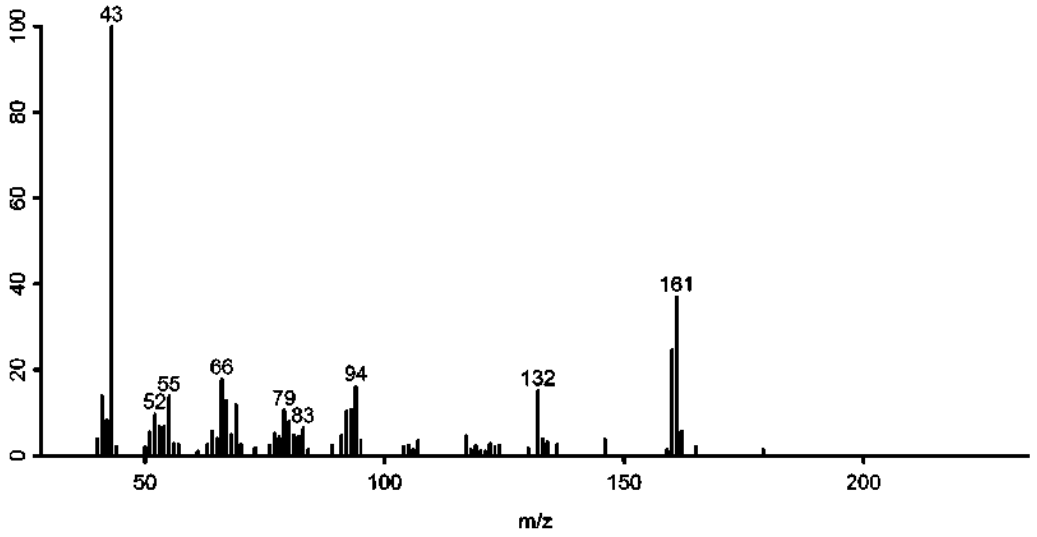

7*Z:* 2-((*Z*)-3’-Hydroxyprop-1’-en-1’-yl)-5-vinyltetrahydrofuran-3-carbonitrile

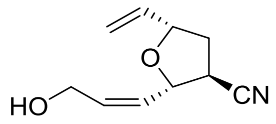

Formuła: **C10H13NO2**

MolWt: **179.0943**

Retention index: **1503**

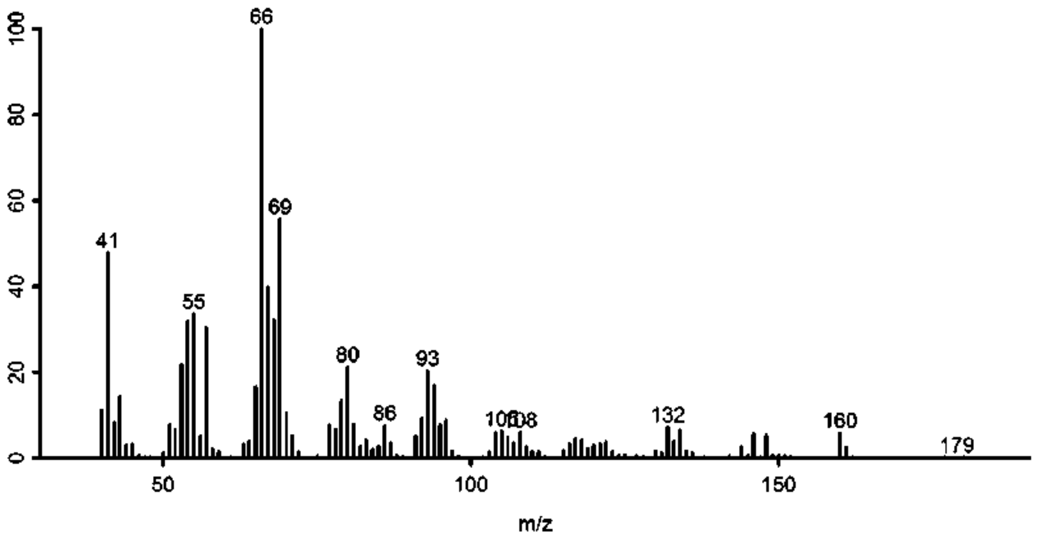

7*E:* 2-((*E*)-3'-Hydroxyprop-1'-en-1'-yl)-5-vinyltetrahydrofuran-3-carbonitrile

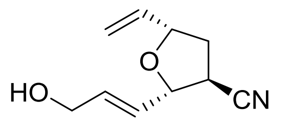

Formuła: **C10H13NO2**

MolWt: **179.0943**

Retention index: **1547**

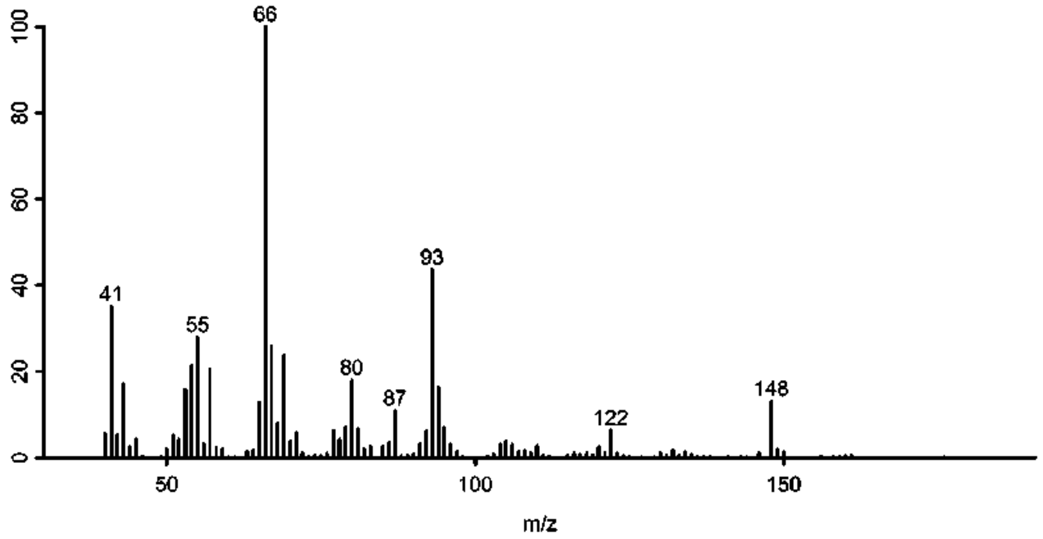

8*Z:* 5-((*Z*)-3'-Hydroxyprop-1'-en-1'-yl)-2-vinyltetrahydrofuran-3-carbonitrile

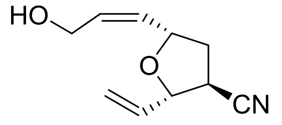

Formuła: **C10H13NO2**

MolWt: **179.0943**

Retention index: **1527**

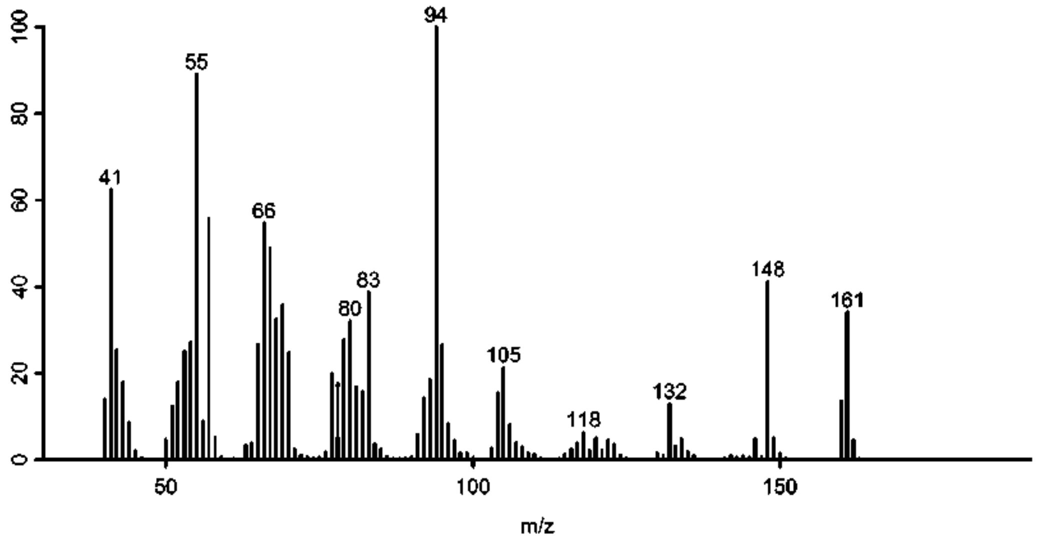

8*E:* 5-((*E*)-3’-Hydroxyprop-1’-en-1’-yl)-2-vinyltetrahydrofuran-3-carbonitrile

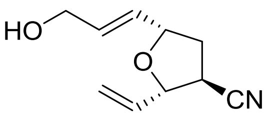

Formuła: **C10H13NO2**

MolWt: **179.0943**

Retention index: **1551**

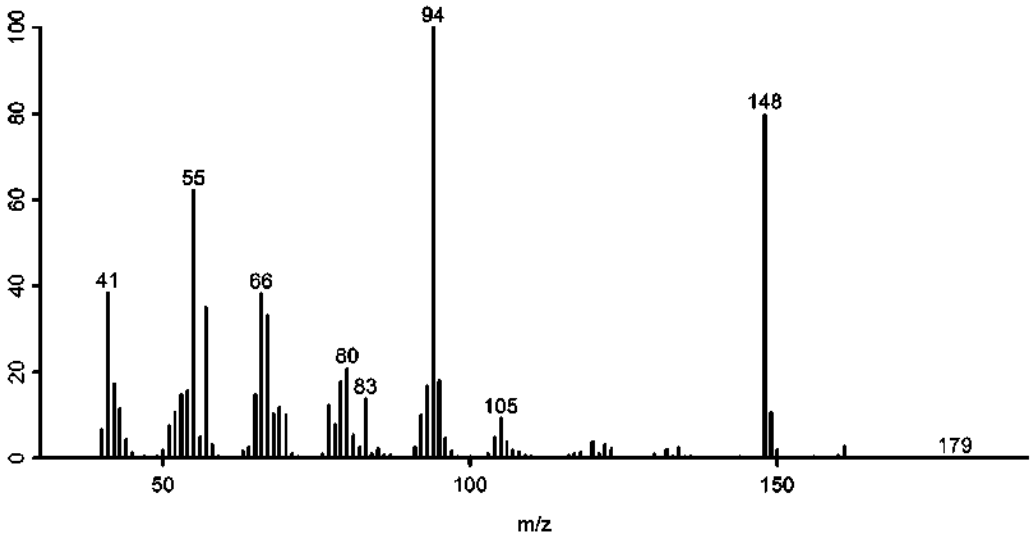

9*Z:* (*Z*)-3’-(3-Cyano-5-vinyltetrahydrofuran-2-yl)allyl acetate

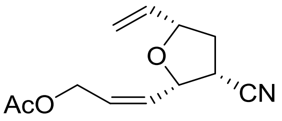

Formuła: **C12H15NO3**

MolWt: **221.1048**

Retention index: **1675**

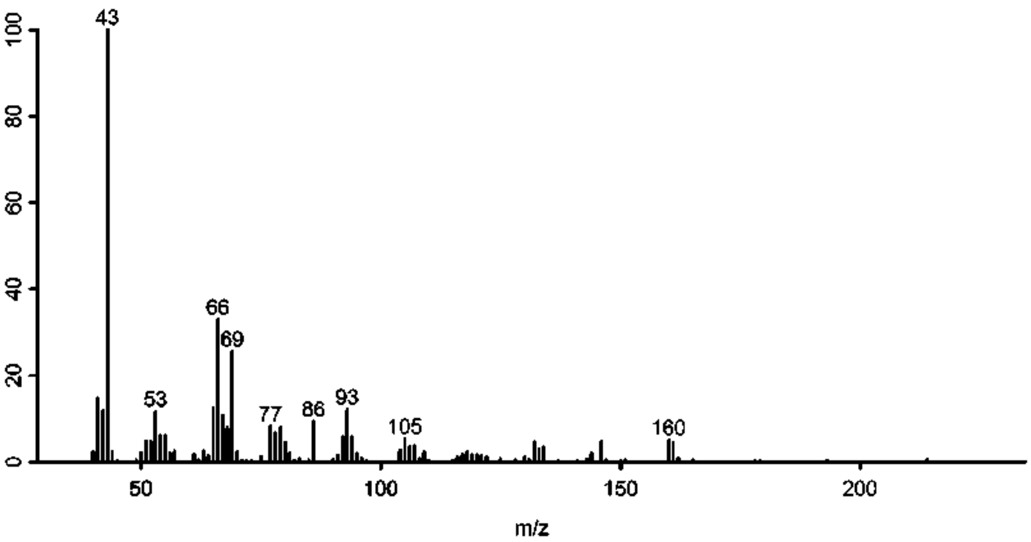

9*E:* (*E*)-3'-(3-Cyano-5-vinyltetrahydrofuran-2-yl)allyl acetate

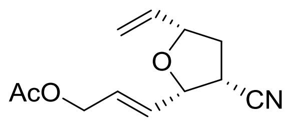

Formuła: **C12H15NO3**

MolWt: **221.1048**

Retention index: **1711**

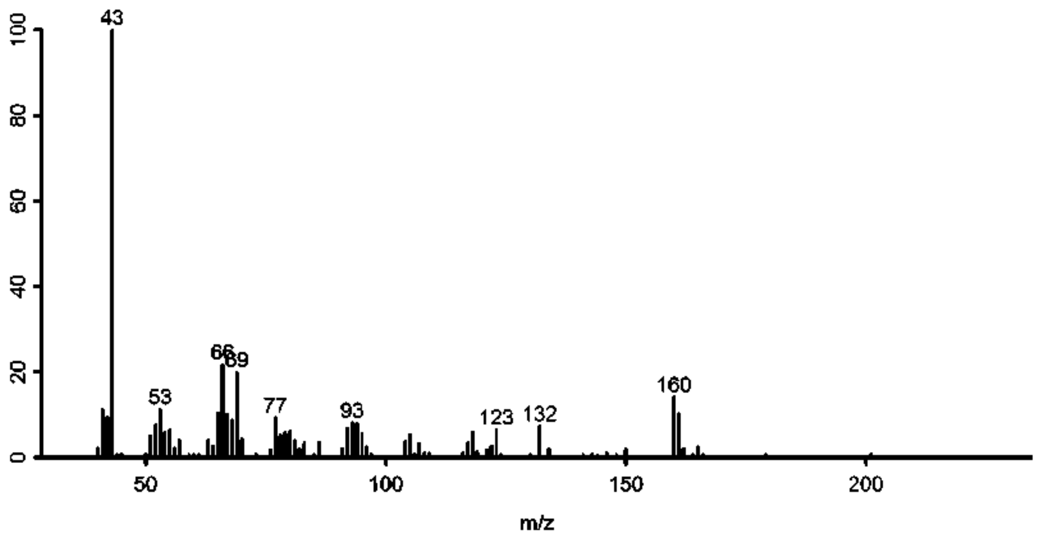

10*Z:* (*Z*)-3'-(4-Cyano-5-vinyltetrahydrofuran-2-yl)allyl acetate

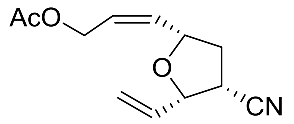

Formuła: **C12H15NO3**

MolWt: **221.1048**

Retention index: **1713**

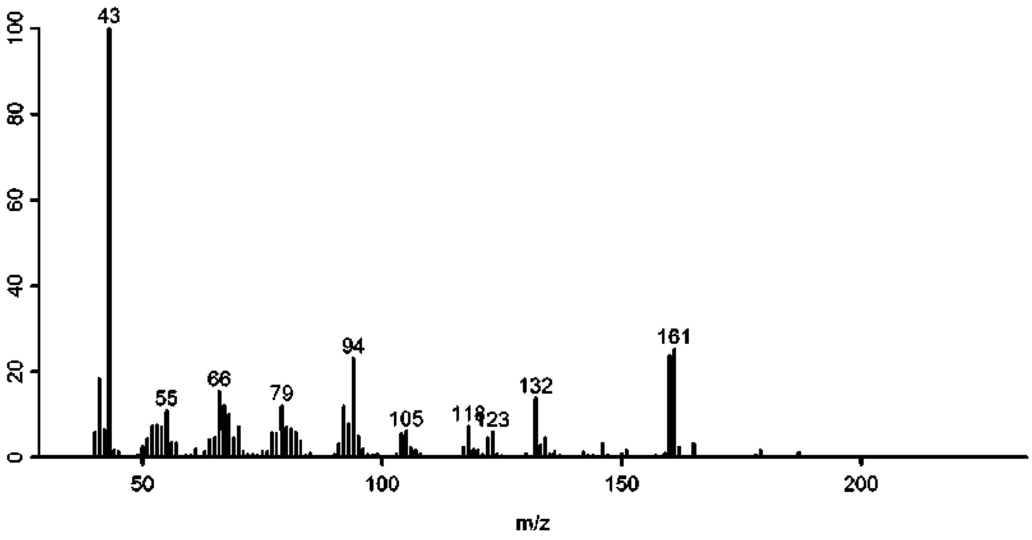

10*E:* (*E*)-3’-(4-Cyano-5-vinyltetrahydrofuran-2-yl)allyl acetate

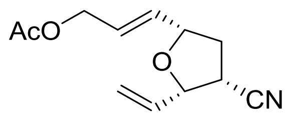

Formuła: **C12H15NO3**

MolWt: **221.1048**

Retention index: **1738**

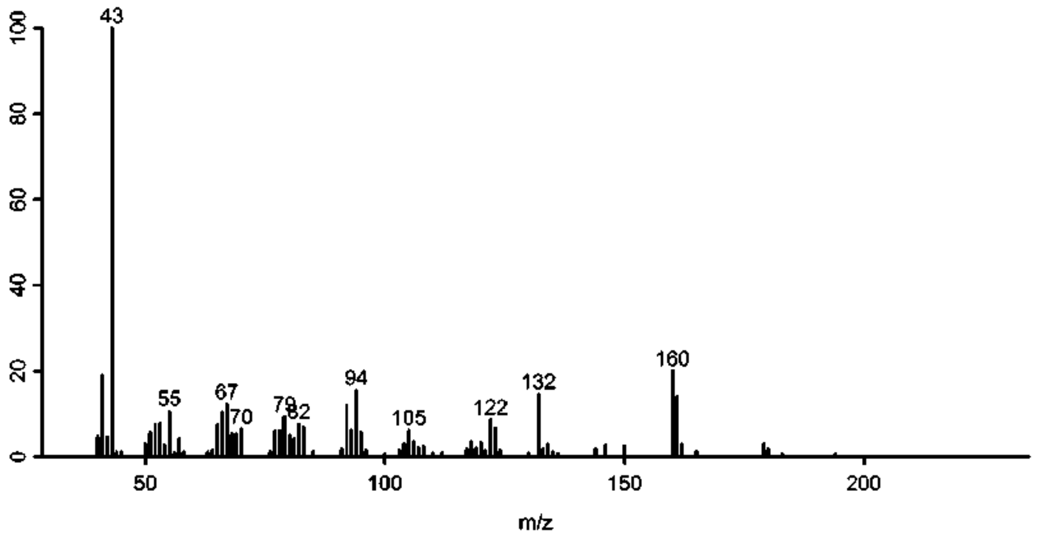

11*Z:* 2-((*Z*)-3’-Hydroxyprop-1’-en-1’-yl)-5-vinyltetrahydrofuran-3-carbonitrile

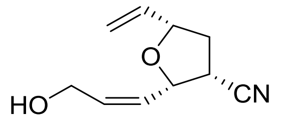

Formuła: **C10H13NO2**

MolWt: **179.0943**

Retention index: **1574**

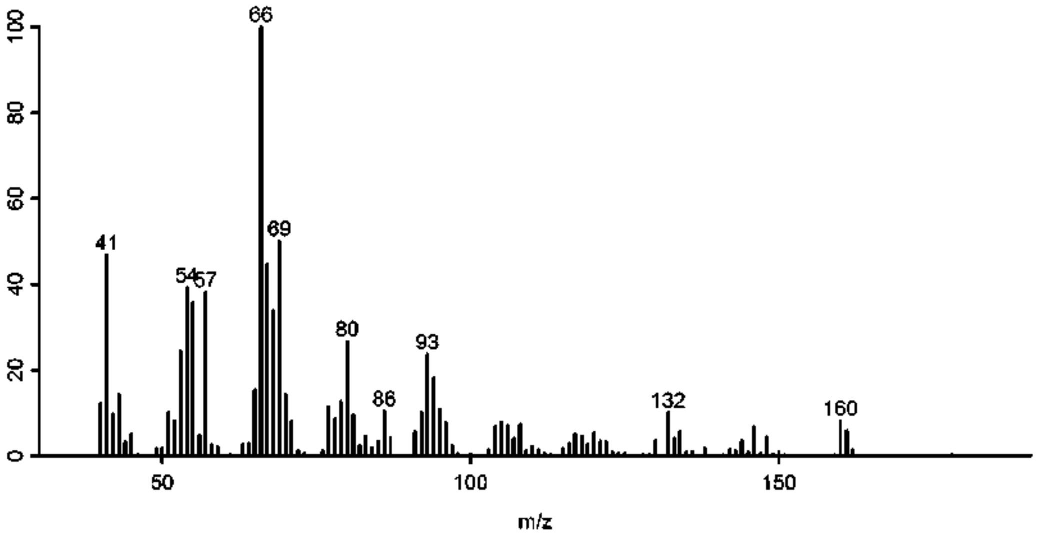

11*E*: 2-((*E*)-3'-Hydroxyprop-1'-en-1'-yl)-5-vinyltetrahydrofuran-3-carbonitrile

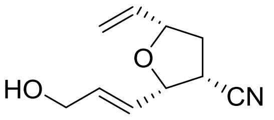

Formuła: **C10H13NO2**

MolWt: **179.0943**

Retention index: **1582**

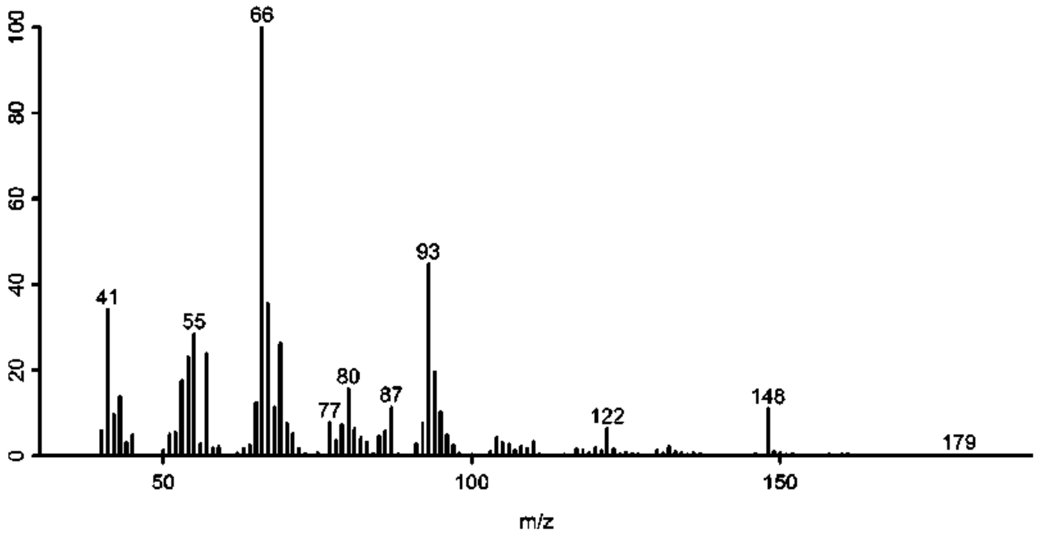

12*Z*: 5-((*E*)-3'-Hydroxyprop-1'-en-1'-yl)-2-vinyltetrahydrofuran-3-carbonitrile

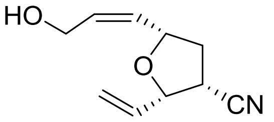

Formuła: **C10H13NO2**

MolWt: **179.0943**

Retention index: **1601**

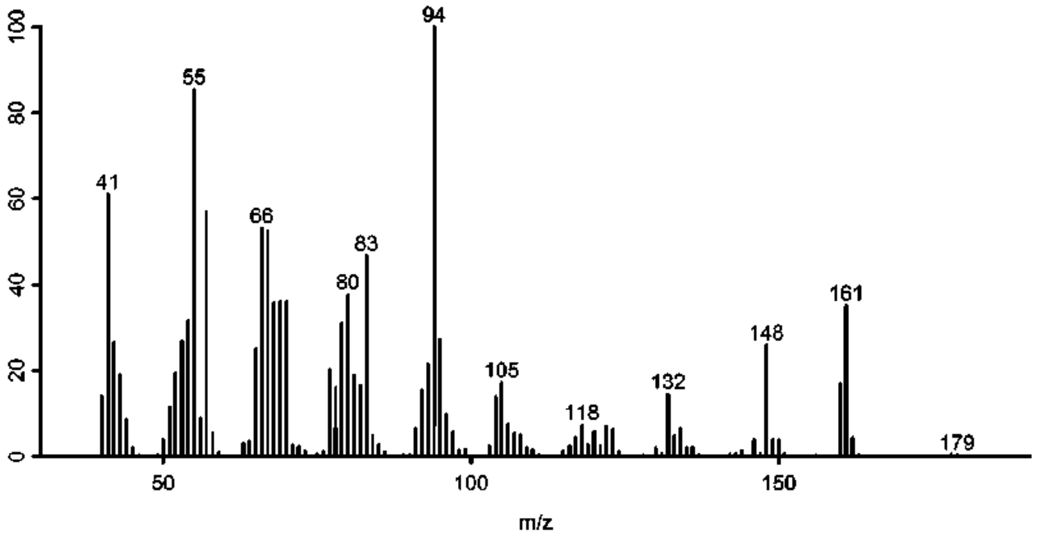

12*E*: 5-((*E*)-3’-Hydroxyprop-1’-en-1’-yl)-2-vinyltetrahydrofuran-3-carbonitrile

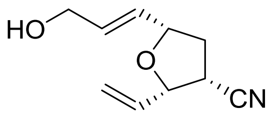

Formuła: **C10H13NO2**

MolWt: **179.0943**

Retention index: **1613**

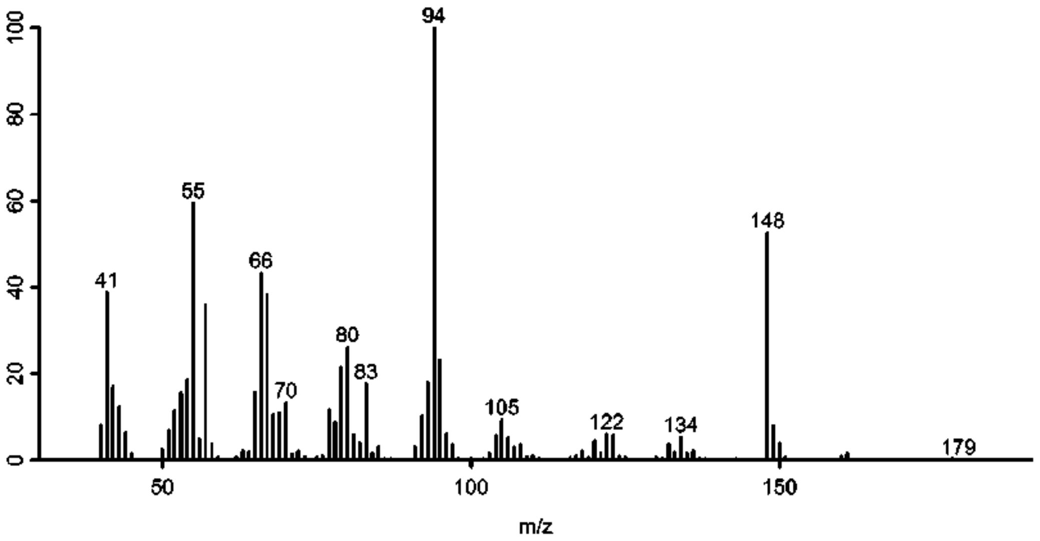

Supplement: File 1 — MS spectra and retention indices of all compounds 5–12. [file Beilstein_J_Org_Chem-11-1893-s001.pdf]
